# Paediatric supracondylar humeral fractures: epidemiology, mechanisms and incidence during school holidays

**DOI:** 10.1007/s11832-014-0577-0

**Published:** 2014-03-19

**Authors:** L. V. Barr

**Affiliations:** Department of Trauma and Orthopaedics, The Ipswich Hospital NHS Trust, Heath Road, Ipswich, IP4 5PD UK

**Keywords:** Paediatric, Supracondylar, Humerus, Fracture, Epidemiology

## Abstract

**Purpose:**

The purpose of this study was to investigate the epidemiology of paediatric patients sustaining supracondylar humeral fractures, to identify common mechanisms of injury and to corroborate the anecdotal evidence that fractures occur more frequently during school holidays.

**Methods:**

All paediatric patients who presented to the accident and emergency department with a supracondylar distal humerus fracture over the 3-year period from 1 July 2008 to 30 June 2011 were included in the study. Data were collected from the electronic medical records and radiology picture archiving and communication system (PACS) regarding age at injury, sex, Gartland type, date of injury, mechanism and management. The dates of all school holidays during the study period were obtained from the local education authority website.

**Results:**

A total of 159 patients were identified, with a median age of 6 years 1 month (range 1 year to 14 years 4 months); 53 % of patients were male. The 155 extension-type injuries comprised 46, 28 and 26 % Gartland I, II and III fractures, respectively. Sixty-five patients (41 %) were treated operatively. Six patients had either neurological and/or vascular complications; however, none had any long-term neurological compromise and none required vascular surgical intervention. The mechanism of injury was recorded in 118 cases, the majority (37 %) of which were sustained during falls from play equipment. Among the patients, 115 were of school age. The weekly incidence during school holidays was significantly higher than that during term-time (1.16 vs. 0.60, *p* = 0.0005).

**Conclusions:**

This study demonstrates the epidemiology of paediatric supracondylar fractures managed at a district general hospital over a 3-year-period. This work supports the long-standing anecdotal evidence that play equipment carries a high risk of injury and that the incidence of supracondylar fractures is significantly higher during school holidays.

## Introduction

Supracondylar humeral fractures are the most common fractures in children under 7 years old [1]. They make up around 15 % of all paediatric fractures. The vast majority are extension type, resulting from a fall onto an outstretched hand, where the elbow is hyperextended, the olecranon is driven into the olecranon fossa and the anterior humeral cortex fails in tension. The pull of triceps tends to displace the distal fragment posteriorly and proximally. Neurovascular complications are reported in 5–19 % of displaced fractures [2, 3], due to the close proximity of structures such as the brachial artery and the anterior interosseous nerve. However, most nerve injuries are a neurapraxia and recover without further intervention [3].

There is anecdotal evidence that there is an increase in the incidence of supracondylar fractures during school holidays, and that falls from play equipment such as monkey bars and trampolines commonly cause these injuries. No study has yet been carried to corroborate this.

The aims of this study were:

1. To compare the incidence of supracondylar fractures during term-time and during holidays in children of school age.
2. To determine the population characteristics, mechanisms of injury, grades of fractures according to Gartland [4] and incidence of any neurovascular complications over a 3-year period at a large district general hospital.

The identification of common mechanisms and times of injury may serve to improve safety and contribute to primary prevention in the future.

## Patients and methods

This retrospective study included all patients aged under 16 years old who had upper limb radiographs over the 3-year period from 1 July 2008 to 30 June 2011 and who were diagnosed with a supracondylar fracture. Data were collected from the electronic medical records, trauma database and the radiology picture archiving and communication system (PACS) regarding age, sex, side, date of injury, Gartland type, mechanism, presence of any neurovascular compromise and management. Those patients for whom incomplete data were available were excluded from the study.

The dates of all school holidays during the study period were obtained from the local education authority website. All school-aged children at the time of injury were identified and the weekly incidence of fractures in this cohort was calculated during the school holidays and during term-time.

## Results

**Fig. 1:**
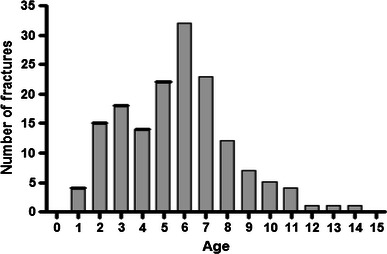
Bar chart showing age distribution of all supracondylar humeral fractures during the study period

Following application of the exclusion criteria, data were analysed for 159 patients, with a median age of 6 years 1 month (range 1 year to 14 years 4 months) (Fig. 1).

Eighty-four patients (53 %) were male and 59 % fractures occurred on the left side. There were no bilateral injuries. There were four flexion-type injuries (3 %), two of which were sustained during falls from a horse. The 155 (97 %) extension-type injuries comprised 46, 28 and 26 % Gartland type I, II and III fractures, respectively.

Sixty-five patients (41 %) were treated operatively: all flexion-type and Gartland type III fractures underwent closed/open reduction + K wiring. Twenty-one (48 %) of the Gartland II fractures required manipulation under a general anaesthetic +/− K wire (Table 1).

**Table 1:** The management and sex distribution of each of the Gartland types

	Number	M:F	Management
I	71 (46 %)	33:38	All conservative in POP
II	44 (28 %)	25:19	23 conservative in POP 21 MUA +/− K wire
III	40 (26 %)	22:18	All MUA +/− K wire
Flexion	4	2:2	3 open reduction + K wire 1 MUA

A total of six patients had documented neurovascular compromise, all of which had sustained Gartland III fractures. Two patients developed an ulnar nerve palsy post-operatively: one underwent surgical exploration, where the nerve was found to be intact and in the other case, the medial wire was removed at 2 weeks. Both patients had full recovery of ulnar nerve function within 2 months of injury. There were two isolated nerve injuries at presentation: a radial nerve neurapraxia which was managed conservatively and fully recovered by 3 months, and a median nerve injury which underwent direct repair at 6 months post-injury and had a good recovery. Two children had signs of vascular compromise: one had an intermittent white pulseless hand—on presentation, the hand was pink with a palpable pulse. However, it became white and pulseless when the elbow was flexed and placed into a backslab. The pulse returned upon removal of the backslab. During manipulation in theatre, it again became white and pulseless; this fully resolved following open reduction of the fracture. The other child had a pink pulseless hand together with median and ulnar nerve palsies—the pulse returned when the fracture was reduced. This patient later underwent exploration and neurolysis of the median and ulnar nerves at 4 weeks post-injury, with a full recovery by 3 months.

**Fig. 2:**
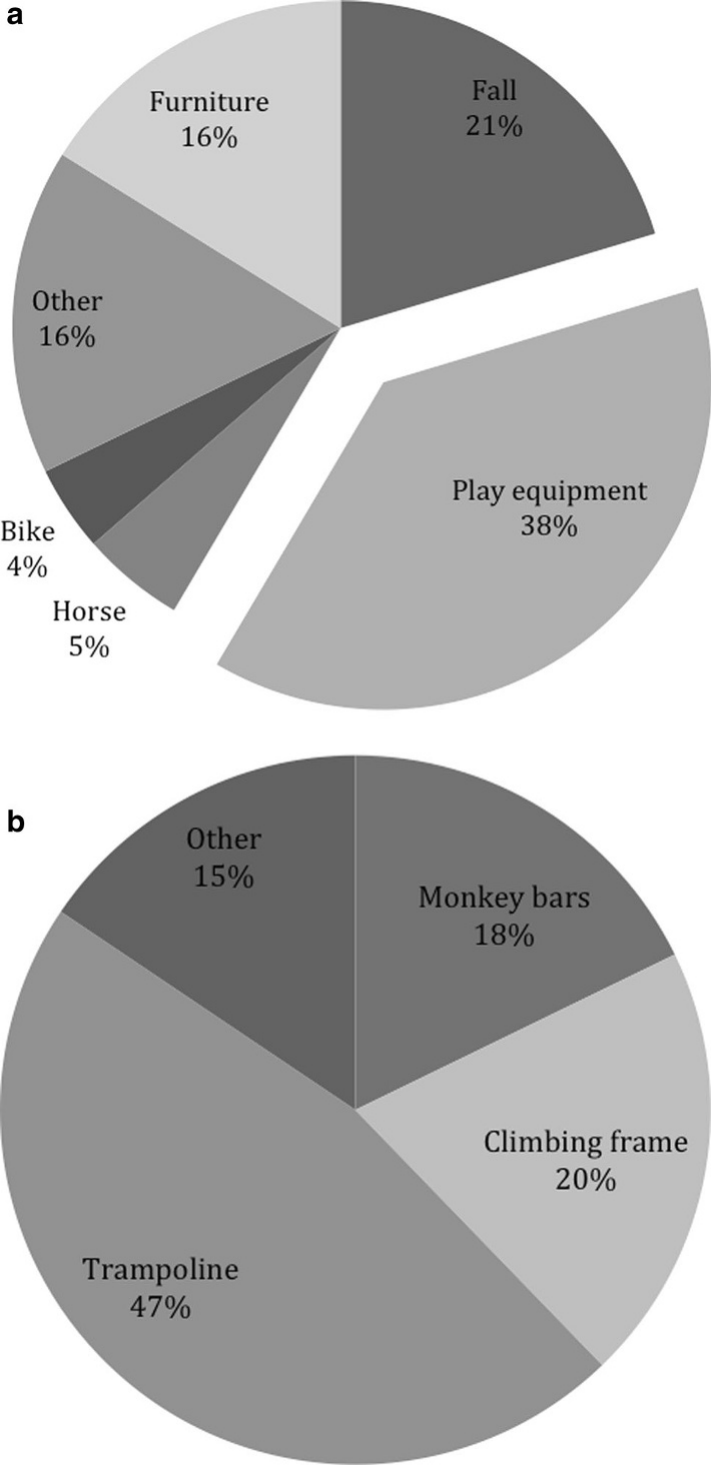
Pie charts showing **a** mechanism of injury, and **b** proportions of each type of playground equipment involved

The mechanism of injury was recorded for 118 (74 %) patients. The majority (38 %) of fractures were sustained during falls from play equipment, almost half of which involved a trampoline (Fig. 2a, b). Sixteen percent of fractures occurred following falls off furniture, such as chairs, sofas or beds; these tended to be among younger children.

**Fig. 3:**
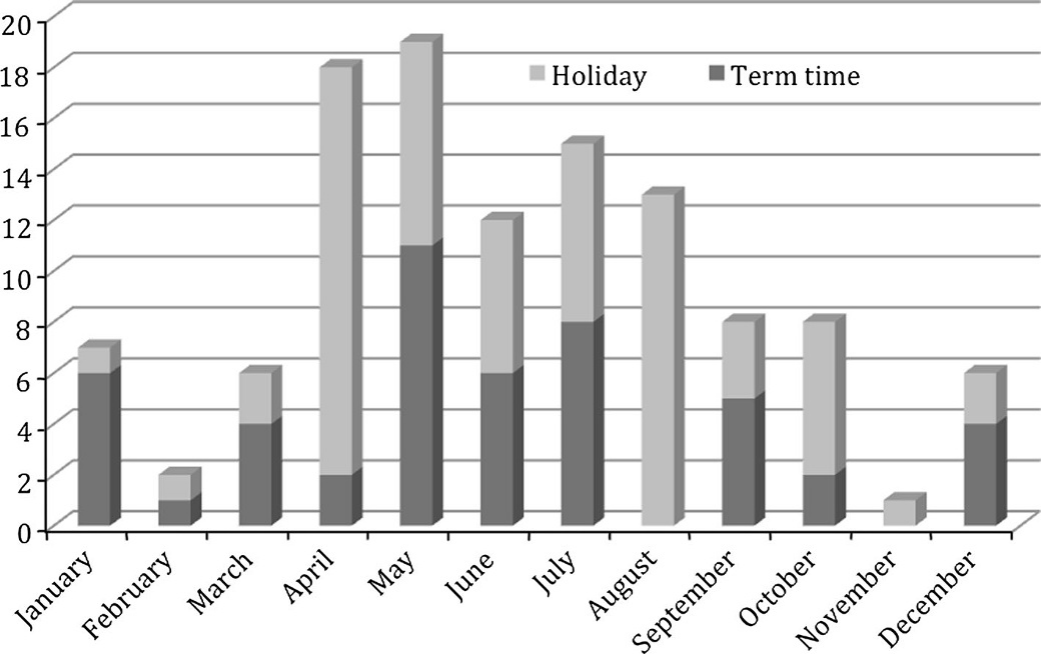
Monthly frequency of supracondylar humeral fractures during term time and holiday time

There were 115 patients identified in the school-aged cohort, with a median age of 6 years 10 months. Forty-four fractures occurred during school holidays and 71 occurred during term-time (49 on school day, and 22 during term-time weekends). The weekly incidence of supracondylar fractures during school holidays was significantly higher than that during term-time (1.16 vs. 0.60, *p* = 0.0005, χ^2^ test). Further, taking into account term-time weekends, there remained a significant increase in the daily incidence of fractures during school holidays (0.15/day) compared to school days (0.08/day) and term-time weekends (0.10/day), *p* = 0.0263. Within the holiday periods, the incidence during the spring and summer was significantly higher than during autumn and winter (Fig. 3).

## Discussion

As in previous studies, there is a peak incidence of supracondylar fractures at around 6 years of age [3, 5], with a predominance of boys being affected [3, 6, 7]. There was a predominance of type I fractures in this population, which is consistent with some previous reports [8], but in contrast to some others [6, 9]. This variability in the rates of type I fractures may reflect either differences in activity levels between different populations—less high-energy activities in the current population—or increased radiological diagnosis of undisplaced fractures.

Of the 84 displaced extension-type fractures, there was documented neurological deficit in five patients (6 %), three of which were noted on presentation. This incidence of nerve injuries is slightly lower than other reports in the literature [3, 8–11]; however, as this is a retrospective notes-based study, the apparent discrepancy may be due to the lack of documentation rather than a true lower incidence.

Few studies have looked at the mode of injury in supracondylar humeral fractures; however, there is much anecdotal evidence suggesting a high incidence of fractures following falls off playground equipment such as monkey bars and trampolines. These results confirm such beliefs, with 38 % of fractures associated with playground equipment. A large proportion (16 %) also occurred following falls off furniture, which tended to be among younger pre-school children, as similarly reported by Farnsworth et al. [12]. While we cannot stop children from playing, the primary prevention of supracondylar fractures may be aided by targeting the playground environment. The introduction of softer landing surfaces beneath play equipment, lower heights, increased adult supervision and an improvement in the overall safety may have significant effects on the incidence of supracondylar fractures, as shown by Park et al. [13].

This study further corroborates the anecdotal evidence that there is an increase in the incidence of supracondylar fractures during school holidays. Within the cohort of school-aged children, the weekly rate of fractures during school holidays is almost twice that of the term-time rate (1.16 vs. 0.60). Unfortunately, little can be done to reduce the higher incidence during holiday times, other than preventative measures in the home and in playgrounds, as mentioned above.

## Conclusion

This study demonstrates the epidemiology of paediatric supracondylar fractures managed at a district general hospital over a 3-year period. The population in this study reflects those in the published literature. The age range, sex distribution, side predominance and rate of neurovascular injury are similar.

The results provide evidence that play equipment such as trampolines, monkey bars and climbing frames carry a high risk of injury and suggests that further guidance may be needed with regards to playground safety. As expected, the incidence of supracondylar humeral fractures in children is significantly higher during school holidays, in particular the summer holidays, and supports the long-standing anecdotal evidence.
